# Effects of Magnesium and Calcium Cations on Antibiotic Susceptibility of *Pseudomonas aeruginosa*

**DOI:** 10.3390/antibiotics15090860

**Published:** 2026-09-03

**Authors:** Hongyu Wang, Yasuhiko Irie

**Affiliations:** Biology Division, Department of Physics, Chemistry, and Biology, Linköping University, 58183 Linköping, Sweden

**Keywords:** *Pseudomonas aeruginosa*, antibiotics, magnesium, calcium, tobramycin, ciprofloxacin

## Abstract

**Background/Objectives:** *Pseudomonas aeruginosa* is an opportunistic Gram-negative bacterium and a major cause of healthcare-associated infections worldwide, exhibiting intrinsic antimicrobial resistance and readily acquiring novel resistance mechanisms. Divalent cations such as Mg^2+^ and Ca^2+^ in the culture environment can influence bacterial antibiotic susceptibility. This study evaluated the impact of varying concentrations of Mg^2+^ and Ca^2+^ on the antibiotic susceptibility of *P. aeruginosa* to tobramycin and ciprofloxacin under both planktonic and biofilm growth modes. **Methods:** The study determined the minimum inhibitory concentration (MIC), minimum bactericidal concentration (MBC), and minimum biofilm eradication concentration (MBEC) to compare alterations in antibiotic susceptibility across different ion conditions and bacterial growth patterns. **Results:** Our findings suggest that elevated concentrations of Mg^2+^ and Ca^2+^ significantly modulate bacterial antibiotic susceptibility. **Conclusions:** The results of our study has implications for limitations in standardised antibiotic susceptibility tests and to therapeutic strategies in cation-rich environments such as cystic fibrosis airways.

## 1. Introduction

*Pseudomonas aeruginosa* is frequently associated with a diverse range of healthcare-associated infections, including ventilator-associated pneumonia, bacteraemia, urinary tract infections, and non-healing wound infections, particularly in immunocompromised individuals [1]. Its clinical significance is particularly pronounced in cystic fibrosis (CF) patients, where *P. aeruginosa* establishes persistent biofilm infections, substantially elevating patient morbidity and mortality rates [2]. *P. aeruginosa* is difficult to eradicate because it combines substantial intrinsic tolerance with a strong capacity to evolve and acquire additional resistance during treatment [3]. Consequently, conventional antimicrobial strategies can become insufficient.

Bacteria can exist in two primary growth states: “planktonic” (freely suspended cells in liquid environments) or biofilm (highly organised communities embedded in an extracellular matrix) [4]. Bacteria within biofilms typically exhibit substantially higher antimicrobial tolerance compared to planktonic counterparts [5]. Early investigations demonstrated that biofilm-associated bacteria can exhibit 100–1000-fold greater tolerance to antimicrobials than planktonic cells [5]. This enhanced tolerance is attributed to multiple factors, including restricted antibiotic diffusion within the biofilm matrix, reduced cellular metabolic activity, and the presence of persister cells within biofilms [6].

In clinical management of *P. aeruginosa* infections, tobramycin and ciprofloxacin are two frequently employed antimicrobial agents [7]. Tobramycin, an aminoglycoside antibiotic, exerts bactericidal activity primarily through binding to the bacterial 30S ribosomal subunit, disrupting protein synthesis by causing the production of erroneous or toxic polypeptides [8]. Aminoglycoside uptake into bacterial cells is dependent on active transport mechanisms and therefore largely contingent upon cell membrane permeability and electrochemical gradients [9]. Ciprofloxacin, a fluoroquinolone antibiotic, functions by inhibiting bacterial DNA gyrase and topoisomerase IV, enzymes essential for DNA replication and transcription [10]. Despite their efficacy in treating *P. aeruginosa* infections, bacteria develop resistance through multiple mechanisms, including reduced membrane permeability, efflux pump overexpression, and target site mutations [11].

Divalent cations, especially Mg^2+^ and Ca^2+^, play pivotal roles in maintaining the structural integrity of the Gram-negative bacterial outer membrane (OM). The OM of *P. aeruginosa* is composed of lipopolysaccharide (LPS), wherein the negatively charged phosphate groups on lipid A require coordination by Mg^2+^ and Ca^2+^ to form intermolecular ionic bridges that stabilise LPS arrangement and maintain membrane integrity [12]. These ionic bridges reduce OM fluidity and permeability, serving as a critical barrier against antibiotic penetration. Consequently, variations in environmental divalent cation concentrations directly alter the degree of OM stabilisation, thereby modulating antibiotic penetration efficiency [13]. Furthermore, Mg^2+^ and Ca^2+^ participate in regulating OM protein expression, LPS modification, and bacterial stress responses. For example, Mg^2+^ can influence the composition of OM proteins and modulate bacterial susceptibility to membrane-active antimicrobial agents [14]. More importantly, divalent cations are critical in biofilm formation. They establish electrostatic cross-links with polysaccharides and extracellular DNA in the extracellular polymeric matrix, rendering biofilm structures more compact and stable [15]. Given that biofilm-associated bacteria typically exhibit antibiotic tolerance substantially exceeding that of planktonic cells [6], the regulation of biofilm architecture by ion concentrations may further influence antibiotic diffusion and bactericidal efficacy within biofilms.

Although several previous studies have investigated the effects of divalent cations on bacterial antibiotic susceptibility [16,17,18,19], most have focused on single growth conditions or limited ion concentration ranges. Systematic studies examining broad gradients of Mg^2+^ and Ca^2+^ concentrations across multiple growth modes remain scarce. In this study, we used *P. aeruginosa* as our model system to simultaneously assess the impact of cation concentrations on antibiotic susceptibility under both planktonic and biofilm growth conditions using the standardised protocols provided by the European Committee on Antimicrobial Susceptibility Testing (EUCAST) as guidance. Given that variations in cation concentrations and complex bacterial growth states exist in certain medically relevant physiological environments, such as in CF lungs [20,21,22,23,24], elucidating how these environmental factors influence antibiotic susceptibility holds important clinical and microbiological significance.

## 2. Result

### 2.1. Effects of Divalent Cations on Antibiotic Susceptibility in Planktonic Cultures

**Figure 1:**
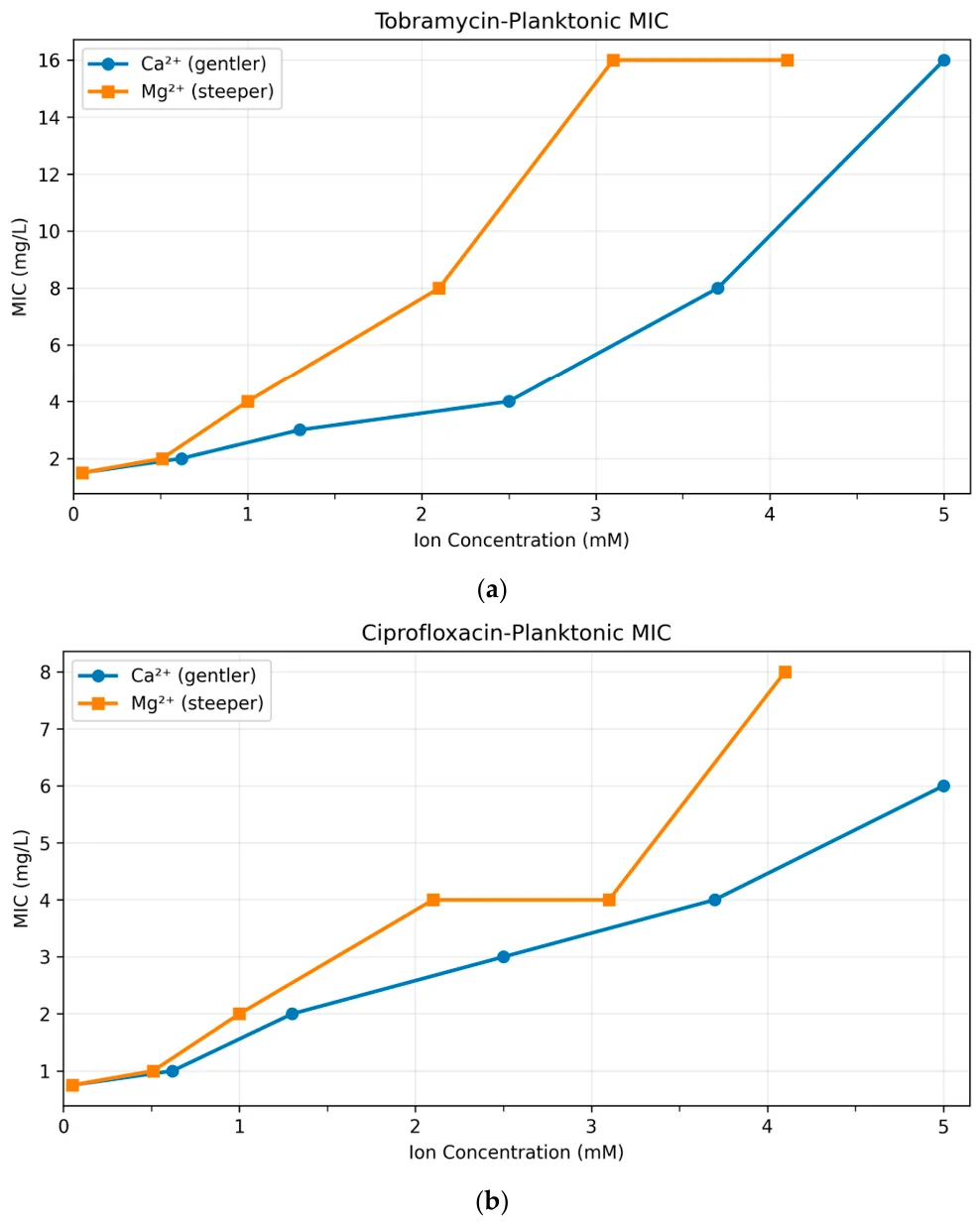
Effects of Ca^2+^ (0–5.0 mM) and Mg^2+^ (0–4.1 mM) on planktonic MIC values for (**a**) tobramycin and (**b**) ciprofloxacin.

Under this study’s experimental conditions, elevated Ca^2+^ and Mg^2+^ concentrations markedly decreased planktonic bacterial susceptibility to both antibiotics. Although the maximum fold changes induced by both cations were similar (approximately 10-fold), their concentration gradients differed (Mg^2+^ 0–100 mg/L, corresponding to 0–4.1 mM; Ca^2+^ 0–200 mg/L, corresponding to 0–5.0 mM), which may reflect differences in how each cation modulates antibiotic susceptibility (Figure 1 and Table 1). When Mg^2+^ was fixed at 0 mg/L, increasing Ca^2+^ from 0 to 200 mg/L (0–5.0 mM) increased the tobramycin minimum inhibitory concentration (MIC) from 1.5 to 16 mg/L and the ciprofloxacin MIC from 0.75 to 6 mg/L. When Ca^2+^ was fixed at 0 mg/L, increasing Mg^2+^ from 0 to 100 mg/L increased tobramycin MIC from 1.5 to 16 mg/L and the ciprofloxacin MIC from 0.75 to 8 mg/L, indicating clear dose–response trends for both ions.

To quantify these trends, linear regression on log_2_-transformed MIC confirmed significant dose–response relationships when the other cation was held at 0 mg/L for ciprofloxacin (Ca^2+^ effect at Mg^2+^ = 0: slope = 0.590 log_2_ units per mM, R^2^ = 0.94, *p* = 0.00119; Mg^2+^ effect at Ca^2+^ = 0: slope = 0.795, R^2^ = 0.93, *p* = 0.00189) and for tobramycin (Ca^2+^ effect at Mg^2+^ = 0: slope = 0.664, R^2^ = 0.99, *p* = 4.47 × 10^−5^; Mg^2+^ effect at Ca^2+^ = 0: slope = 0.898, R^2^ = 0.94, *p* = 0.00162). To compare the strength of Ca^2+^ vs. Mg^2+^ effects under the “other cation = 0 mM” condition, a combined model including a concentration × cation-type interaction term indicated significantly steeper Mg^2+^ slopes than Ca^2+^ slopes for both ciprofloxacin (*p* = 0.00439) and tobramycin (*p* = 0.00348). Finally, when testing whether the dose–response slope changed in the presence of the EUCAST CAMHB standard level of the other cation (Mg^2+^ = 12.5 mg/L, 0.51 mM or Ca^2+^ = 23 mg/L, 0.57 mM), no significant slope changes were detected for ciprofloxacin (Ca^2+^ effect: *p* = 0.147; Mg^2+^ effect: *p* = 0.0768) or tobramycin (Ca^2+^ effect: *p* = 0.882; Mg^2+^ effect: *p* = 0.730), suggesting no strong evidence that the standard level of the other cation altered the linear dose–response slope within the tested range.

### 2.2. Effects of Divalent Cations on Antibiotic Susceptibility in Biofilm Cultures

**Figure 2:**
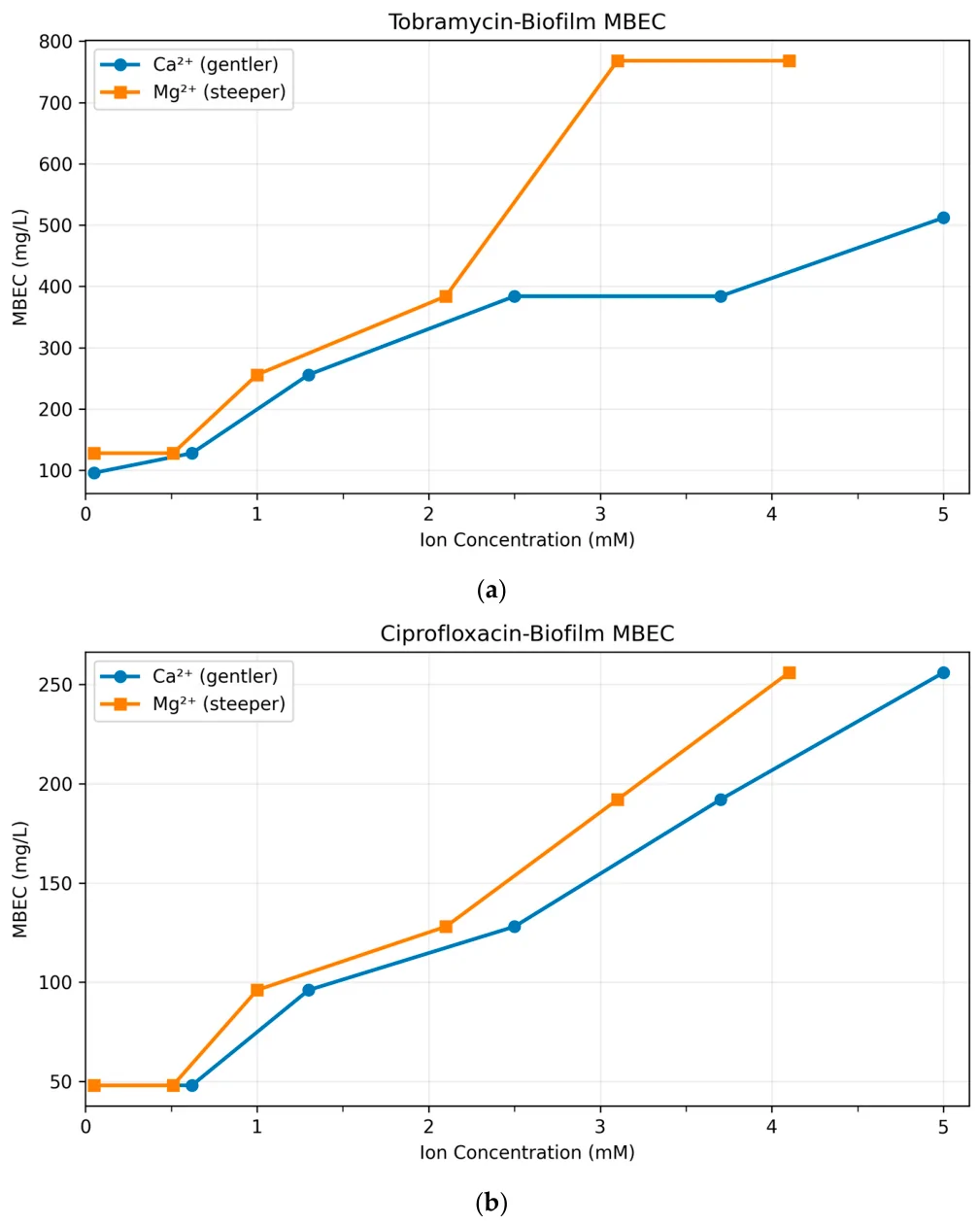
Effects of Ca^2+^ (0–5.0 mM) and Mg^2+^ (0–4.1 mM) on biofilm MBEC values of (**a**) tobramycin and (**b**) ciprofloxacin.

Biofilm-associated bacteria demonstrated substantially higher antibiotic tolerance than their planktonic counterparts, and increasing divalent cation concentrations further increased tolerance, as reflected by higher MBEC values (Figure 2 and Table 2). When Mg^2+^ was fixed at 0 mM, increasing Ca^2+^ from 0 to 200 mg/L (0–5.0 mM) increased ciprofloxacin MBEC from 48 to 256 mg/L and tobramycin MBEC from 96 to 512 mg/L. When Ca^2+^ was fixed at 0 mg/L, increasing Mg^2+^ from 0 to 100 mg/L (0–4.1 mM) increased ciprofloxacin MBEC from 48 to 256 mg/L and tobramycin MBEC from 128 to 768 mg/L, consistent with a strong concentration-dependent reduction in biofilm susceptibility.

To quantify these effects, linear regression on log_2_-transformed MBEC showed significant dose–response relationships when the other cation was held at 0 mM for ciprofloxacin (Ca^2+^ effect at Mg^2+^ = 0: slope = 0.512 log_2_ units per mM, R^2^ = 0.95, *p* = 0.00101; Mg^2+^ effect at Ca^2+^ = 0: slope = 0.616, R^2^ = 0.95, *p* = 0.00114) and for tobramycin (Ca^2+^ effect at Mg^2+^ = 0: slope = 0.466, R^2^ = 0.85, *p* = 0.00871; Mg^2+^ effect at Ca^2+^ = 0: slope = 0.711, R^2^ = 0.93, *p* = 0.00186). Comparing Ca^2+^ vs. Mg^2+^ dose–response slopes under the “other cation = 0 mM” condition, the concentration × ion-type interaction term supported significantly steeper Mg^2+^ slopes than Ca^2+^ slopes for ciprofloxacin (*p* = 0.0147) and tobramycin (*p* = 0.00768). Finally, when assessing whether the presence of the EUCAST CAMHB standard level of the other ion altered the dose–response slope, no significant slope changes were observed for ciprofloxacin (Ca^2+^ effect: *p* = 0.612; Mg^2+^ effect: *p* = 0.689) or tobramycin (Ca^2+^ effect: *p* = 0.874; Mg^2+^ effect: *p* = 0.724), indicating that within these data the standard level of the other cation did not measurably change the linear dose–response slope.

### 2.3. Comparison of Planktonic and Biofilm Growth Modes

Across all ion conditions and both antimicrobials, biofilm-associated bacteria demonstrated significantly higher absolute resistance levels than their planktonic counterparts. Using EUCAST CAMHB as the baseline, tobramycin baseline values were planktonic MIC = 2 mg/L and biofilm MBEC = 128 mg/L, yielding a biofilm-to-planktonic fold ratio of 128/2 = 64-fold for tobramycin and 48/1 = 48-fold for ciprofloxacin.

**Figure 3:**
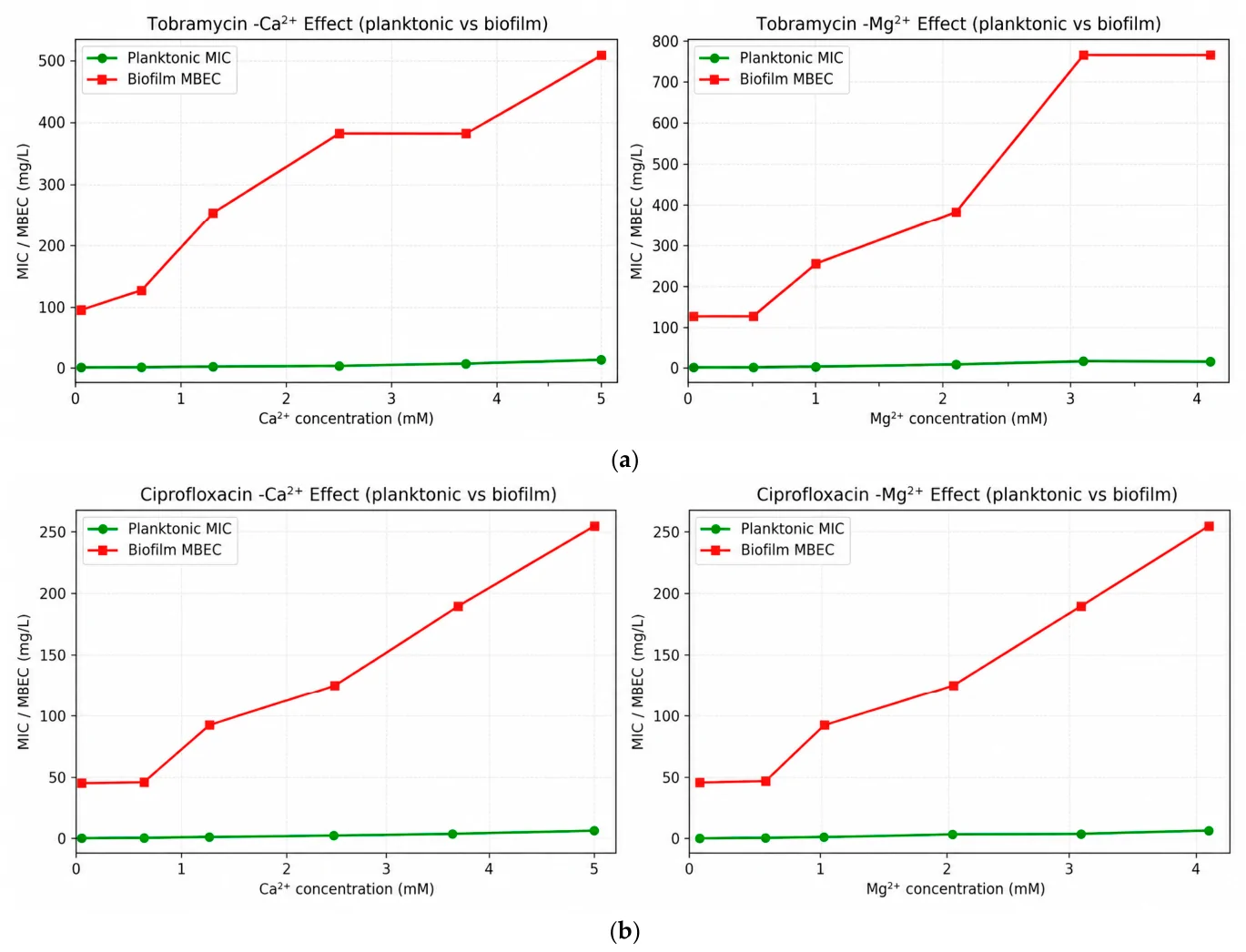
Comparison of the effects of Ca^2+^ and Mg^2+^ on antibiotic susceptibilities between planktonic and biofilm growth modes. (**a**) Tobramycin MIC (planktonic) vs. MBEC (biofilm) across Ca^2+^ and Mg^2+^ gradients, showing higher tolerance in biofilms (48–96× at matched cation conditions). (**b**) Ciprofloxacin MIC (planktonic) vs. MBEC (biofilm) across Ca^2+^ and Mg^2+^ gradients, showing higher tolerance in biofilms (32–64× at matched cation conditions).

When comparing growth modes across different concentration gradients (Figure 3), biofilm MBEC values consistently exceeded planktonic MIC values at matched cation concentrations. For example, at a Ca^2+^ concentration of 100 mg/L (2.5 mM): planktonic tobramycin MIC = 4 mg/L, while biofilm MBEC = 384 mg/L (96-fold difference between modes). At a Mg^2+^ concentration of 50 mg/L (2.1 mM): planktonic resistance = 8 mg/L versus biofilm resistance = 384 mg/L (48-fold increase). Statistical trends in both modes were highly significant (Pearson correlation coefficient *r* and ANOVA: *p* < 0.01). This demonstrates that divalent cation-induced resistance operates via distinct mechanisms in planktonic versus biofilm contexts, with biofilms achieving absolute protection levels far exceeding those of the planktonic counterparts.

Although biofilms exhibited significantly higher baseline and cation-induced resistance, the qualitative response pattern (resistance increasing with cation concentration) remained consistent across modes. The distinction was quantitative: at matched cation conditions, biofilms achieved 32–96-fold greater absolute tolerance than planktonic cells, underscoring the dominant role of biofilm architecture in fostering antibiotic tolerance (Figure 3).

### 2.4. Comparative Ion-Specific Differential Effects: Mg^2+^ Versus Ca^2+^

Both Mg^2+^ and Ca^2+^ produced concentration-dependent decreases in tobramycin and ciprofloxacin efficacies. For planktonic bacteria, both Ca^2+^ (0–5.0 mM) and Mg^2+^ (0–4.1 mM) produced clear concentration-dependent increases in endpoints. To quantify the cation-specific differential effect, we compared dose–response slopes by fitting a linear model to log_2_-transformed MIC versus cation concentration. For tobramycin, MIC increased from 1.5 → 16 mg/L across both gradients, but Mg^2+^ showed a steeper log_2_ slope than Ca^2+^ (Mg: 0.898 vs. Ca: 0.664 log_2_ units per mM), consistent with Mg^2+^ achieving the same plateau over a narrower concentration range. For ciprofloxacin, MIC rose from 0.75 → 6 mg/L across the Ca^2+^ gradient and 0.75 → 8 mg/L across the Mg^2+^ gradient, again with a steeper Mg^2+^ slope (Mg: 0.795 vs. Ca: 0.590 log_2_ units per mM). The concentration endpoint association was strong for both cations (correlation between concentration and log_2_ (MIC): Ca^2+^ *r* = 0.995/Mg^2+^ *r* = 0.967 for tobramycin; Ca^2+^ *r* = 0.972/Mg^2+^ *r* = 0.964 for ciprofloxacin).

For biofilm bacteria, using the same log_2_ (MBEC) per mM slope comparison, Mg^2+^ drove a faster escalation of tobramycin MBEC than Ca^2+^ (Mg^2+^ slope 0.711 vs. Ca^2+^ 0.466 log_2_ units per mM). Consistent with this, MBEC increased from 96 → 512 mg/L across the Ca^2+^ gradient and from 128 → 768 mg/L across the Mg^2+^ gradient (other cation fixed at 0 mM). For ciprofloxacin MBEC, the two gradients reached the same ceiling under the other cation = 0 mM (48 → 256 mg/L for both Ca^2+^ and Mg^2+^), with only a modest slope difference (Mg^2+^ 0.616 vs. Ca^2+^ 0.512 log_2_ units per mM). Cation-specific differences for ciprofloxacin in biofilms appear more strongly under CAMHB background (Mg^2+^ fixed at 0.51 mM vs. Ca^2+^ fixed at 0.57 mM), where the Ca^2+^ gradient reaches a higher maximum MBEC than the Mg^2+^ gradient.

Based on these fitted dose–response models, we estimated the cation concentration associated with a predicted two-fold increase in the antibiotic susceptibility endpoint (which we refer to in this paper as “C_2_-fold”). For planktonic cultures, the estimated C_2_-fold concentrations for tobramycin were 1.11 mM (27.1 mg/L) for Mg^2+^ and 1.51 mM (60.4 mg/L) for Ca^2+^, while those for ciprofloxacin were 1.26 mM (30.6 mg/L) and 1.69 mM (67.9 mg/L), respectively (Table 3). For biofilm cultures, the corresponding C_2_-fold concentrations were 1.41 mM (34.2 mg/L) for Mg^2+^ and 2.15 mM (86.0 mg/L) for Ca^2+^ for tobramycin, and 1.62 mM (39.5 mg/L) and 1.95 mM (78.3 mg/L) for ciprofloxacin, respectively (Table 3). Thus, under the tested conditions, the model predicted a lower cation concentration for Mg^2+^ than for Ca^2+^ to produce a two-fold increase in MIC or MBEC.

Therefore, both Mg^2+^ and Ca^2+^ function as pro-tolerance factors, yet their kinetic characteristics are growth mode dependent. Planktonic cells exhibit comparable MIC responses to both cations despite differing concentration ranges, whereas biofilm cells preferentially escalate tolerance via Mg^2+^ over Ca^2+^. These distinctions underscore the complexity of cation–antibiotic–biofilm interactions and suggest that therapeutic strategies may require consideration of cation-specific resistance mechanisms in distinct cellular contexts (Figure 1 and Figure 2 and Table 1 and Table 2).

## 3. Discussion

An important finding of this study is that, under both planktonic and biofilm conditions, elevated concentrations of Mg^2+^ and Ca^2+^ both decrease the effectiveness of the two different classes of antibiotics we tested: the aminoglycoside tobramycin and the fluoroquinolone ciprofloxacin. In cystic fibrosis airways, elevated Ca^2+^ and Mg^2+^ concentrations have been reported in sputum [24], where *P. aeruginosa* commonly forms biofilms [25,26,27]. Similar elevations have also been reported in other pulmonary diseases, including bronchiectasis [28], where *P. aeruginosa* is also a major colonising pathogen [29]. These concentrations represent several-fold increases compared to healthy individuals [30]. Furthermore, extracellular cation concentrations are known to change during skin wound healing processes, with magnesium notably increasing during early wound healing processes [20,21,31] and elevated levels of calcium frequently seen in non-healing wounds [32]. Therefore, the concentration ranges investigated here provide a clinically relevant experimental context for examining how divalent cations may influence antibiotic susceptibility in *P. aeruginosa*.

The mechanisms behind these phenomena remain unclear but could be attributed to ionic stabilisation of the OM. In earlier studies, polycationic agents such as aminoglycosides were observed to increase OM permeability, while Mg^2+^ antagonised this permeabilisation [12,13]. This is consistent with divalent cation cross-bridging of adjacent LPS molecules, which reduces permeability and thereby limits antibiotic access [33]. This provides a direct physical basis for competition between divalent cations and cationic antibiotics at the OM surface [34]. In addition to envelope stabilisation, fluoroquinolones such as ciprofloxacin are known to form complexes with divalent cations (Mg^2+^/Ca^2+^) [35]. Such physicochemical interactions may influence drug accumulation and antibacterial activity independently of bacterial physiology. However, recent analyses [36] emphasise that while complexation is well-documented in vitro, it may not solely account for reduced antibacterial activitiy in vivo.

Across the tested ranges, both Ca^2+^ and Mg^2+^ increased antibiotic tolerance in a concentration-dependent manner. When one cation was absent, Mg^2+^ showed a consistently steeper dose–response trend than Ca^2+^, but adding the EUCAST CAMHB “standard level” of the other cation did not produce a clear slope change. Within the limits of this dataset and model, the combined effects appear approximately additive or sub-additive rather than synergistic under these conditions. The steeper Mg^2+^ slopes in both planktonic and biofilm assays suggest that Mg^2+^ may more potently modulate tolerance pathways. The lower model-derived C_2_-fold concentrations for Mg^2+^ than for Ca^2+^ were consistent with the steeper Mg^2+^ dose–response slopes observed in both planktonic and biofilm conditions. However, these values should be interpreted as concentration-response estimates within the experimental range rather than as clinical therapeutic thresholds. Possible mechanisms include enhanced extracellular polysaccharide expression, efflux pump regulation, and differences in bioavailability or intracellular chelation.

A parsimonious biological interpretation is that, in Gram-negative cells, divalent cation effects can converge on partially overlapping envelope-related effects such as charge shielding and stabilisation at anionic surface sites in addition to cation-triggered stress-adaptive responses [37,38]. At the phenotypic level, the diminishing marginal returns are more likely to occur once one cation has already substantially altered the key step, because further increases in the other cation produce progressively smaller additional effects. Under biofilm conditions, Ca^2+^ is known to additionally shape extracellular matrix mechanics and architecture by interacting with negatively charged components [39,40]. If Ca^2+^ establishes a dominant matrix-/architecture-linked protective state in biofilms, the incremental contribution from Mg^2+^ may be comparatively limited, making additive or sub-additive combined effects more likely, consistent with the lack of strong interaction signals observed for biofilm endpoints in this study. These hypotheses provide a biological explanation for why Mg^2+^ achieved comparable resistance plateaus over a narrower concentration range than Ca^2+^.

The findings of this study offer multiple insights into *P. aeruginosa* infections. In CF airways, elevated Ca^2+^ and Mg^2+^ levels, originating from inflammatory exudates, host cells, and degraded matrix components, are commonly detected in sputum and bronchoalveolar lavage samples from CF patients [24]. The present data suggest that such divalent cation-enriched microenvironments are predicted to amplify biofilm tolerance up to 64-fold for tobramycin and 16-fold for ciprofloxacin. These effects may partly account for the occasionally observed discrepancy between in vitro susceptibility results and clinical efficacy of inhaled aminoglycosides or fluoroquinolones in CF patients. For example, analysis of the European tobramycin nebuliser solution trial reported that clinical and bacteriological responses to inhaled tobramycin were independent of baseline MIC, suggesting that conventional resistance metrics may underestimate effectiveness when very high airway concentrations are achieved [41]. In addition, a retrospective analysis of CF pulmonary exacerbations treated with tobramycin plus ceftazidime found no correlation between isolate susceptibility and post-treatment FEV1 improvement [42]. Our results support renewed interest in ion-chelating strategies as adjunctive therapies. Chelators such as EDTA and EGTA have been shown to disrupt biofilms in both in vitro and in vivo models [43,44,45]. These data highlight the value of formulating antimicrobial regimens based not only on the pathogen and its resistance genes but also on the physicochemical environment of the infection site, including divalent cation levels, by guiding personalised treatments, for example through sputum ion profiling or analyses of other common biofilm-colonised medical surfaces such as catheter effluents. The results of this study also justify considering similar inspections using different antibiotics across various pathogenic species, and furthermore, other cations such as zinc and iron.

## 4. Materials and Methods

### 4.1. Bacterial Strain and Culture Media

*P. aeruginosa* PAO1 strain was used as the model organism in this study [46]. The strain was initially cultivated on Lysogeny Broth (LB) Lennox agar plates overnight at 37 °C to obtain isolated colonies. For antibiotic susceptibility experiments, two types of Mueller–Hinton media were used:

1. Cation-adjusted Mueller–Hinton broth (CAMHB; BBL™ Mueller–Hinton II; Fisher Scientific, Göteborg, Sweden), containing approximately 10–12.5 mg/L (corresponding to 0.4–0.5 mM) Mg^2+^ and 20–25 mg/L (corresponding to 0.5–0.6 mM) Ca^2+^, was used as the standard medium for antibiotic susceptibility testing. Mg^2+^ and Ca^2+^ solutions were prepared to match the EUCAST standard cation concentrations for CAMHB (specified in mg/L). For analysis and reporting, all ion concentrations were converted to mM.
2. Cation-free Mueller–Hinton broth (cation-free MHB; Difco™ standard formulation; Fisher Scientific) was used for experimental manipulation of ion concentrations. For the purpose of this paper, the “cation-free” medium was defined as containing 0 mM supplemented Mg^2+^/Ca^2+^.

In addition, Mueller–Hinton agar plates (MHA) were used for colony enumeration. All media were prepared according to the manufacturers’ instructions under aseptic conditions. All media were freshly prepared on the day of experimentation and subjected to autoclaving. Divalent cations were supplemented as MgCl_2_ and CaCl_2_. Corresponding stock solutions were prepared in cation-free MHB and sterilised by membrane filtration. Tobramycin and ciprofloxacin stock solutions were also sterilised by membrane filtration and serially diluted to generate the required concentration gradients.

### 4.2. Ion Concentration Conditions

To investigate the effects of Mg^2+^ and Ca^2+^ on antibiotic susceptibility, experiments incorporated an EUCAST standard [47] control group and multiple ion gradient experimental groups. The EUCAST standard control group utilised CAMHB containing approximately 10–12.5 mg/L (corresponding to 0.4–0.5 mM) Mg^2+^ and 20–25 mg/L (corresponding to 0.5–0.6 mM) Ca^2+^. Experimental groups employed cation-free MHB supplemented with MgCl_2_ or CaCl_2_ to establish different concentration gradients:

Mg^2+^ concentration gradients: 0, 12.5, 25, 50, 75, 100 mg/L

(corresponding to 0, 0.51, 1.0, 2.1, 3.1, 4.1 mM)

Ca^2+^ concentration gradients: 0, 25, 50, 100, 150, 200 mg/L

(corresponding to 0, 0.62, 1.3, 2.5, 3.7, 5.0 mM)

The cation-free MHB served as the low-ion control group.

### 4.3. Antibiotic Susceptibility Testing in Planktonic Cultures

**Figure 4:**
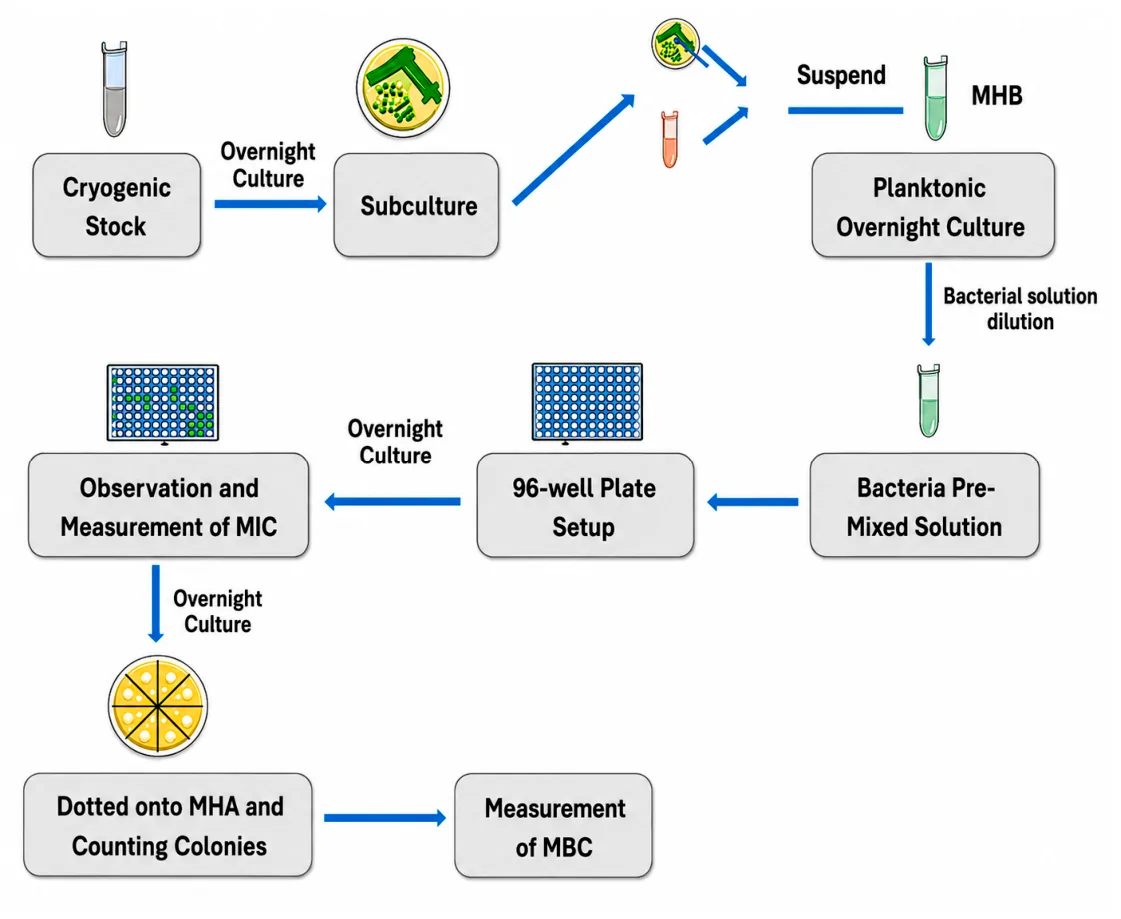
A flow diagram of the steps in the planktonic culture mode process for antimicrobial susceptibility testing using 96-well plates.

Antibiotic susceptibility of planktonic cells was determined using the broth microdilution method according to EUCAST antibiotic susceptibility testing standards. The experimental schematic is shown in Figure 4. Single colonies were inoculated into MHB liquid medium. The bacterial suspension was adjusted to approximately a 0.5 McFarland standard and further diluted to approximately 1 × 10^6^ CFU/mL as the inoculum. In 96-well microtiter plates, 100 µL of medium containing varying antibiotic concentrations was added to each well, followed by 100 µL of bacterial suspension. Negative controls (sterile medium) and positive controls (antibiotic-free bacterial suspension) were included in each assay. Plates were incubated at 37 °C for 18–24 h, after which bacterial growth was assessed by visual inspection of turbidity. The minimum inhibitory concentration (MIC) was defined as the lowest antibiotic concentration that completely inhibited visible bacterial growth. To determine the minimum bactericidal concentration (MBC), 10 µL aliquots from wells showing no visible growth were spot-inoculated onto MHA plates and cultured for 24 h at 37 °C for colony enumeration. The MBC was defined as the lowest antibiotic concentration resulting in a ≥99.9% reduction in bacterial cell counts.

### 4.4. Antibiotic Susceptibility Testing in Biofilm Cultures

**Figure 5:**
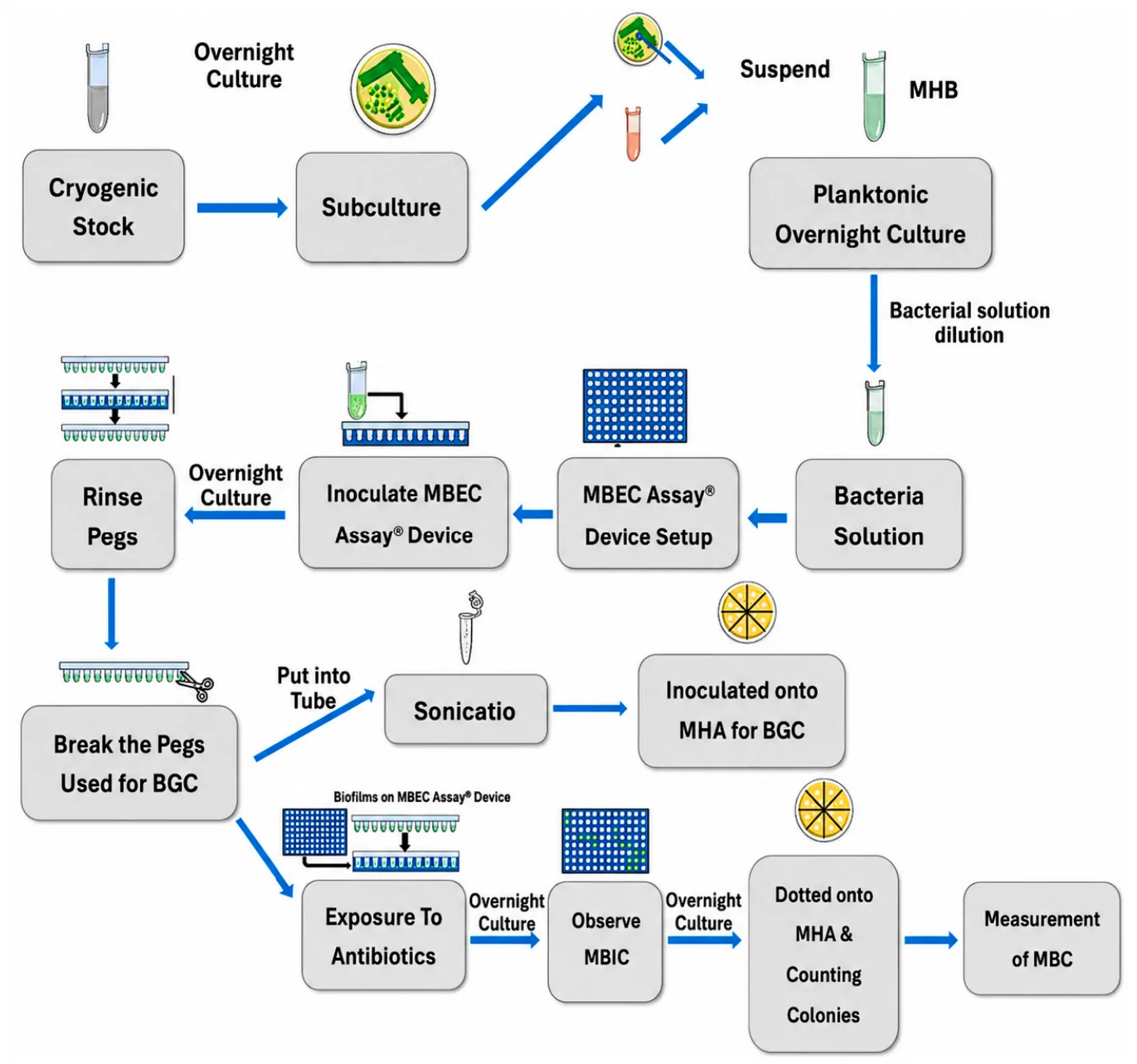
A flow diagram of the steps in the biofilm culture mode process for antimicrobial susceptibility testing using the MBEC Assay^®^ Biofilm Inoculator with a 96-well base.

The experimental schematic for biofilm experiments is shown in Figure 5. Biofilm antibiotic susceptibility was assessed using the MBEC Assay^®^ Biofilm Inoculator system (Innovotech, Edmonton, AB, Canada). Single colonies were inoculated into 3 mL of cation-free MHB and cultured overnight at 37 °C with shaking at 200 rpm. The culture was subsequently diluted to approximately a 0.5 McFarland standard and further diluted to approximately 1 × 10^6^ CFU/mL. The bacterial suspension was added to 96-well plates and covered with a peg lid, then incubated statically at 37 °C for 24 h in cation-free MHB to allow biofilm formation. After incubation, the biofilm was gently rinsed with sterile saline solution to remove planktonic cells. Selected portions of pegs were then removed for the biofilm growth check (BGC), which served as an auxiliary indicator of biofilm biomass. The remaining pegs were then transferred to wells containing media with various antibiotic concentrations and incubated for an additional 24 h at 37 °C for antibiotic exposure. Following antibiotic treatment, the peg lid was transferred to wells containing sterile physiological saline and sonicated for 10 min to dislodge biofilm cells [48]. Subsequently, detached cells were serially diluted, and 10 µL aliquots were inoculated onto MHA plates and cultured for 24 h to enumerate colonies. The minimum biofilm eradication concentration (MBEC) was defined as the lowest antibiotic concentration resulting in ≥99.9% killing of biofilm-associated cells.

Biofilm Growth Check (BGC)

$$Killing\%=\left(1-\frac{{CFU}_{treated}}{{CFU}_{untreated\;control}}\right)\times 100$$

The purpose of recording BGC data in this study was to serve as an auxiliary indicator of overall biofilm growth, biomass, and bacterial load [49]. It helped determine whether the “baseline growth state” of the biofilm changed substantially under different ionic conditions, thereby providing essential context for interpreting susceptibility endpoints such as MBEC (e.g., whether MBEC changes were driven primarily by altered antibiotic tolerance or occurred together with differences in biofilm biomass). In short, BGC functioned more as a “descriptive reference metric for biofilm state” than as a direct susceptibility endpoint itself.

The calculation formula for BGC was:

$$\mathrm{CFU}/\mathrm{peg}\;=\frac{plate\;colony\;count\times dilution\;factor}{0.05mL}$$

derived from the operational guidelines of the MBEC Assay^®^ Biofilm Inoculator (Innovotech).

### 4.5. Statistical Analyses

All experiments were performed in at least independent biological triplicates, with each replicate including technical replicates. The raw dataset is available as Supplementary Material (Table S1). For planktonic assays, MIC was used as the primary outcome, whereas for biofilm assays, MBEC was used as the primary outcome. To account for the stepwise (two-fold dilution) nature of susceptibility endpoints, MIC and MBEC values were log_2_-transformed prior to statistical modelling. Three complementary analyses were performed to address the study questions:

(a) To test whether each ion exerted a dose–response effect when the other ion was held at 0 mM, linear regression was performed with log_2_ (MIC) or log_2_ (MBEC) as the response and ion concentration (mM) as a continuous predictor, and the significance of the slope term was evaluated.

$${log}_{2}(Y)=\beta_{0}+\beta_{1}\;C$$

Y: MIC (planktonic) or MBEC (biofilm)

C: Ion concentration (Mg^2+^ 0–100 mg/L, corresponding to 0–4.1 mM, Ca^2+^ 0–200 mg/L, corresponding to 0–5.0 mM)

β_0_: Intercept

β_1_: Main effect slope of concentration (how much log_2_ (Y) changes for every 1 mM increase

(b) To determine whether the dose–response slopes differed between Ca^2+^ and Mg^2+^ under the “other ion = 0 mM” condition, a combined linear model including an interaction term (concentration × ion type) was fitted, where the interaction term tested slope differences.

$${log}_{2}(Y)=\beta_{0}+\beta_{1}\;C+\beta_{2}\;I\;+\beta_{3}\;(C\;\times \;I)$$

I: Categorical indicator of ion type; Ca^2+^ is the reference group (I = 0), Mg^2+^ is the other group (I = 1).

C × I: Interaction term, indicating whether the concentration effect changes with ion type.

(c) To assess ion–ion interaction, models including an interaction term between concentration and the “other ion level” group (0 mM vs. EUCAST CAMHB standard level; Mg^2+^ = 12.5 mg/L, corresponding to 0.51 mM or Ca^2+^ = 23 mg/L, corresponding to 0.57 mM) were fitted to determine whether the dose–response slope changed in the presence of the standard level of the other ion.

$${log}_{2}(Y)=\beta_{0}+\beta_{1}\;C+\beta_{2}\;G\;+\beta_{3}\;(C\;\times \;G)$$

G: Group variable, indicating the level of another ion.

For example, when analysing a Ca^2+^ gradient: G = 0 indicates Mg^2+^ = 0 mg/L; G = 1 indicates Mg^2+^ = 12.5 mg/L, corresponding to 0.51 mM (EUCAST standard).

C × G: Interaction term, indicating whether the concentration slope changes due to the other ion level.

(d) For interpretive purposes, the concentration associated with a predicted two-fold increase in MIC or MBEC relative to the model-predicted value at zero cation concentration (“C_2_-fold”) was estimated from the fitted regression slope calculated using the formula shown below.

$$C_{2-fold}=\frac{{log}_{2}\left(1\right)}{\beta_{1}}=\frac{1}{\beta_{1}}$$

Statistical analyses were conducted using GraphPad Prism software (version 11.0.1). Statistical significance was defined as *p* < 0.05.

### 4.6. Figure Creation and Artwork

The figures for this paper were created using Python (version 3.14) in Visual Studio Code (version 1.118) and GraphPad Prism.

## Figures and Tables

**Table 1:** Planktonic antibiotic susceptibility summary. Each condition was tested in three independent experiments; in most cases, replicate MIC/MBEC readouts were identical and therefore overlap in the plots. In addition, MIC/MBEC values are measured on a discrete two-fold dilution scale, so changes smaller than one dilution step would not be visually detectable.

CAMHB (EUCAST Standard Ca^2+^ = 0.57 mM, Mg^2+^ = 0.51 mM)							
**Tobramycin** **(mg/L)**	**MIC**	2					
	**MBC**	4					
**Ciprofloxacin** **(mg/L)**	**MIC**	1					
	MBC	2					
**Mg^2+^ = 0**	**Ca^2+^ (mM)**	**0**	**0.62**	**1.3**	**2.5**	**3.7**	**5.0**
Tobramycin (mg/L)	MIC	1.5	2	3	4	8	16
	MBC	3	4	6	8	16	48
Ciprofloxacin (mg/L)	MIC	0.75	1	2	3	4	6
	MBC	1.5	2	4	6	8	12
**Mg^2+^ = 0.51 mM**	**Ca^2+^ (mM)**	**0**	**0.62**	**1.3**	**2.5**	**3.7**	**5.0**
Tobramycin (mg/L)	MIC	2	2	4	6	12	16
	MBC	4	4	6	12	32	64
Ciprofloxacin (mg/L)	MIC	1	1	2	3	4	6
	MBC	2	2	4	6	8	12
**Ca^2+^ = 0**	**Mg^2+^ (mM)**	**0**	**0.51**	**1.0**	**2.1**	**3.1**	**4.1**
Tobramycin (mg/L)	MIC	1.5	2	4	8	16	16
	MBC	3	4	8	16	32	48
Ciprofloxacin (mg/L)	MIC	0.75	1	2	4	4	8
	MBC	1.5	2	3	4	8	12
**Ca^2+^ = 0.57 mM**	**Mg^2+^ (mM)**	**0**	**0.51**	**1.0**	**2.1**	**3.1**	**4.1**
Tobramycin (mg/L)	MIC	2	2	4	8	16	16
	MBC	4	4	6	16	32	48
Ciprofloxacin (mg/L)	MIC	1	1	2	4	4	8
	MBC	2	2	4	6	8	10

**Table 2:** MBEC values of tobramycin and ciprofloxacin under different Ca^2+^ (0–5.0 mM) and Mg^2+^ (0–4.1 mM) concentrations.

CAMHB (EUCAST Standard Ca^2+^ = 0.57 mM, Mg^2+^ = 0.51 mM)							
**Tobramycin** **(mg/L)**	**MBEC**	**128**					
**Ciprofloxacin** **(mg/L)**		**48**					
**BGC (CFU/Peg)**		**4.28 × 10^8^**					
**Mg^2+^ = 0**	**Ca^2+^ (mM)**	**0**	**0.62**	**1.3**	**2.5**	**3.7**	**5.0**
Tobramycin (mg/L)	MBEC	96	128	256	384	384	512
Ciprofloxacin (mg/L)		48	48	96	128	192	256
BGC (CFU/Peg)		4.02 × 10^8^	3.88 × 10^8^	3.74 × 10^8^	2.92 × 10^8^	2.41 × 10^8^	1.62 × 10^8^
**Mg^2+^ = 0.51 mM**	**Ca^2+^ (mM)**	**0**	**0.62**	**1.3**	**2.5**	**3.7**	**5.0**
Tobramycin (mg/L)	MBEC	128	128	384	384	512	768
Ciprofloxacin (mg/L)		48	48	96	128	256	384
BGC (CFU/Peg)		4.61 × 10^8^	4.35 × 10^8^	3.22 × 10^8^	3.80 × 10^8^	4.92 × 10^8^	5.90 × 10^8^
**Ca^2+^ = 0**	**Mg^2+^ (mM)**	**0**	**0.51**	**1.0**	**2.1**	**3.1**	**4.1**
Tobramycin (mg/L)	MBEC	128	128	256	384	768	768
Ciprofloxacin (mg/L)		48	48	96	128	192	256
BGC (CFU/Peg)		5.86 × 10^8^	5.74 × 10^8^	4.72 × 10^8^	3.48 × 10^8^	4.98 × 10^8^	3.46 × 10^8^
**Ca^2+^ = 0.57 mM**	**Mg^2+^ (mM)**	**0**	**0.51**	**1.0**	**2.1**	**3.1**	**4.1**
Tobramycin (mg/L)	MBEC	128	128	256	512	768	768
Ciprofloxacin (mg/L)		48	48	96	128	192	256
BGC (CFU/Peg)		5.14 × 10^8^	5.63 × 10^8^	4.41 × 10^8^	3.47 × 10^8^	5.31 × 10^8^	3.02 × 10^8^

**Table 3:** Cation concentrations required to produce a predicted two-fold increase in MIC (**a**) and MBEC (**b**) (“C_2_-fold”) relative to the baseline condition.

**Planktonic MIC C_2_-fold**			
**Antibiotic**	**Cation**	**β_1_ (log_2_ units/mM)**	**C_2-fold_ (Fold Concentration)**
Tobramycin	Ca^2+^	0.664	1.51 mM (60.4 mg/L)
Tobramycin	Mg^2+^	0.898	1.11 mM (27.1 mg/L)
Ciprofloxacin	Ca^2+^	0.590	1.69 mM (67.9 mg/L)
Ciprofloxacin	Mg^2+^	0.795	1.26 mM (30.6 mg/L)
**Biofilm MBEC C_2_-fold**			
**Antibiotic**	**Cation**	**β_1_ (log_2_ units/mM)**	**C_2-fold_ (Fold Concentration)**
Tobramycin	Ca^2+^	0.446	2.15 mM (86.0 mg/L)
Tobramycin	Mg^2+^	0.711	1.41 mM (34.2 mg/L)
Ciprofloxacin	Ca^2+^	0.512	1.95 mM (78.3 mg/L)
Ciprofloxacin	Mg^2+^	0.616	1.62 mM (39.5 mg/L)

## Data Availability

The raw dataset is available in the Supplementary Materials (Table S1).

## Supplementary Materials

The following supporting information can be downloaded at: https://www.mdpi.com/article/10.3390/antibiotics15090860/s1, Table S1. Raw dataset in this study.
